# Effectiveness of link workers providing social prescribing on health outcomes and costs for adult patients in primary care and community settings. A protocol for a systematic review of the literature.

**DOI:** 10.12688/hrbopenres.12936.2

**Published:** 2020-11-17

**Authors:** Bridget Kiely, Aisling Croke, Eamon O'Shea, Deirdre Connolly, Susan M. Smith

**Affiliations:** 1 Department of General Practice, Royal College of Surgeons in Ireland, Dublin, Ireland; 2 Centre for Economic and Social Research on Dementia, National University of Ireland, Galway, Galway, Ireland; 3 Discipline of Occupational Therapy, Trinity College, Dublin, Ireland

**Keywords:** Link worker, Community navigator, Community health worker, Social prescribing, Primary care, Multimorbidity, Social deprivation, Systematic Review

## Abstract

**Introduction: **The use of link workers for social prescribing and health and social care coordination is increasing, but there is insufficient data to demonstrate their effectiveness or for whom they work best. Multimorbidity is increasing in prevalence and affects those living in deprived areas ten years earlier than affluent areas. This systematic review aims to examine the evidence for the effectiveness and costs of link workers in improving health outcomes. We will also look for evidence for the use of link workers specifically for people living with multimorbidity and in deprived areas.

**Methods: **Databases of published and grey literature will be searched for randomised and non-randomised controlled trials examining use of link workers based in primary care for community dwelling adults compared to usual care. Primary outcomes will be health related quality of life and mental health. Data on costs will be extracted. Studies will be selected for inclusion by title and abstract review by two reviewers. A Preferred Reporting Items for Systematic Reviews (PRISMA) flow diagram will document the selection process. A standardised form will be used to extract data. Data quality will be assessed using the Cochrane Risk of Bias tool for randomised controlled trials, a narrative synthesis will be completed and the GRADE assessment tool used to comment on evidence quality. A meta-analysis of effect size of primary outcomes and subgroup analysis for multimorbidity and social deprivation will be performed if there are sufficient comparable data.

**Conclusion:** This systematic review will give an important overview of the evidence for the use of link workers providing social prescribing and health and social care coordination in primary care. This will help inform intervention development and guide policy makers on whether these interventions are cost effective and which groups stand to benefit most. **Prospero** **registration: **CRD42019134737 (04/07/2019)

## Introduction

Social prescribing is a way of linking people with complex needs to non-medical supports in the community ^1^. Social prescribing aims to improve quality of life by addressing some of the social determinants of health in particular reducing social isolation ^1^. There are different models of social prescribing, which can range from online signposting services to individual support from a link worker to identify and access community resources. The link worker model is most frequently used in the UK ^2^. A link worker is a non-health or social care professional based in primary care practices or community and/or voluntary organisations, who support access to a range of community-based resources and supports for health and social care. They usually, but not always, have some training in behaviour change and an in depth knowledge of local community based resources. They meet with people referred to them, usually through primary care, and work with them to achieve a set of personalised goals. These may be purely psychosocial or may involve support to attend services such as diabetic eye screening or access social welfare benefits. In this way, the link worker role extends beyond social prescribing.

Multimorbidity is defined as two or more chronic health conditions. Multimorbidity is increasing in prevalence ^3^. People experiencing multimorbidity have worse health outcomes, more service utilisation, experience fragmented care and poorer quality of life than those without. There is a known link between the social determinants of health and multimorbidity. People who live in socially deprived areas experience multimorbidity at least ten years before those in the least deprived areas and are more likely to have mental health comorbidities ^3, 4^. This translates into increased consultation rates and more complex psychosocial needs that pose a challenge for primary care in deprived areas.

There is potential that link workers providing social prescribing and supporting health and social care coordination can have an impact on health outcomes and costs for people experiencing multimorbidity, particularly in areas of social deprivation. Despite this potential, the evidence for link workers providing social prescribing and health and social care coordination remains weak, with a recent systematic review of UK interventions identifying very few randomised control trials and concluding that there was a lack of evidence for how, for whom and when social prescribing was effective ^2^. Since then there has been an evaluation of the Glasgow Deep End Link workers intervention, which showed improvements in mental health scores, but not health related quality of life and concluded that further evidence was needed ^5^. There are similar interventions being implemented outside the UK such as the IMPaCT intervention using community health workers to support people with complex needs in areas of deprivation ^6^. A randomised controlled trial showed reduced hospital admissions, but no improvement in the primary outcome, self rated health ^7^. To date there has not been a systematic review that we are aware of that examined link worker interventions internationally, effectiveness in areas of deprivation or for people experiencing multimorbidity.

In this systematic review we aim to examine the evidence of effectiveness of link worker social prescribing interventions internationally and to establish the evidence, if any, for its effectiveness in people experiencing multimorbidity and social deprivation. The evidence on the cost effectiveness of social prescribing is weak, making it difficult to say with any certainty for whom and how well existing schemes work. Studies that have considered economic issues both cost and outcomes, including long-term resource use, will, therefore, be considered in the review. This involves the summary and synthesis of data from relevant health economics studies and the extraction of any data on costs reported in relevant studies. The review will also consider studies that have calculated the social return on investment (SROI) associated with social prescribing.

The review questions are

1. What is the effectiveness of link workers providing social prescribing on improving health outcomes for community dwelling adults?

2. What is the effectiveness of link workers providing social prescribing on improving health outcomes for community dwelling adults with multimorbidity?

3. What is the effectiveness of link workers providing social prescribing on improving health outcomes for adults living in areas of social deprivation?

4. What are the costs and cost effectiveness of link workers providing social prescribing in primary care and community settings?

## Methods

### Eligibility criteria

We will search for randomised controlled trials and non-randomised controlled trials that meet the criteria outlined in the Cochrane Effective Practice and Organisation of Care (EPOC) guidance on study design ^8^ from inception of databases to present, with no language restrictions.

***Participants/population.***

Inclusion: Community dwelling adults, who are not residing in residential or supported care and are attending primary care. Primary care will be generally defined as “care provided by clinicians that are available to treat all common conditions in all age groups and have an ongoing relationship with their patients” ^9^. Participants do not need to have any specific index condition.

Exclusion: Under 18s, those in residential or supported care

***Intervention***

Inclusion: Link workers providing health and social care coordination including social prescribing with a focus on accessing non-medical interventions but also supporting individuals with multimorbidity based on their priorities. Link workers are non-health or social care professionals based in primary care practices or community and/or voluntary organisations, who support access to a range of community-based resources and supports for health and social care. They usually, but not always, have some training in behaviour change and an in depth knowledge of local community based resources. Social prescribing is a mechanism for linking people with non-medical sources of support within the community to improve physical, emotional and mental wellbeing. The model of social prescribing can vary, but for the purposes of this review we are specifically interested in models that include the use of link workers. Link workers may be known by other terms such as community health workers, patient navigators or health facilitators. While all of these work in the area of health, they are generally considered “lay workers” as they have not completed a professional health or social work qualification. Inclusion is based on the function of the role, i.e. supporting people to improve their health and wellbeing through social prescribing and health and social care coordination, recognising that there is a wide range of terms used to describe such roles. For simplicity, the term “link worker” is used in this protocol.

The intervention must involve

- A referral (including self referrals) to a link worker who is based either in a primary care practice or a community or voluntary organisation
- Participants meeting with a link worker face to face at least once
- Determining an individual range of health and social care supports and community activities and resources that the person would be willing to engage with and being offered support and follow up to engage with their chosen supports and activities
- The intervention is designed to be tailored to the individual therefore there are no restrictions on the supports and activities that can be recommended, other than the restrictions based on local availability

Exclusions: Interventions that do not involve a link worker, use volunteers as link workers (as these would not be deemed professionals) or only involve signposting to services will be excluded. Interventions where additional support is being provided by health care professionals such as doctors, nurses, occupational therapists or by social workers will be excluded. Interventions were personal care is provided alongside health and social care coordination such as disability support workers will be excluded as it is not possible to separate the effects of the different components of care.

While a particular index condition such as diabetes or depression may have been the reason for referral, the intervention should be holistic and not condition specific. Interventions focused on improving outcomes for a specific condition only will be excluded. Interventions, which mainly comprise of education and goal setting around disease control, even if they have a small element of social prescribing, will be excluded, as it would not be possible to separate the impact of the different components of the intervention.

The interventions of interest should take place in a primary care or community based setting. Interventions that are secondary care or emergency department based will be excluded. 

***Comparator(s)***

Inclusion: All studies will include a comparator group. This comparator group will be no referral to a link worker or (in the case of before-after studies) before referral to a link worker.

Exclusion: No comparator

***Types of study***

Inclusion: Randomised controlled trials (RCTs) or non-randomised controlled trials (nRCTs) will be included based on the Cochrane EPOC guidance on study eligibility for these designs ^8^.

Economic analysis will include cost studies, cost effectiveness analysis (CES), cost benefit analysis (CBA), cost utility analysis (CUA), willingness to pay (WTP), social return on investment (SROI).

Exclusion: Qualitative and uncontrolled descriptive studies will not be included.

***Setting.*** Primary care will be generally defined as “care provided by clinicians that are available to treat all common conditions in all age groups and have an ongoing relationship with their patients” ^9^. This definition allows for a more flexible interpretation in countries that have different models of healthcare. The intervention should be based in primary care and/or the community. While there may be referrals that originate from secondary care or emergency departments, this should not be the main setting for the intervention.

The definition of social deprivation is debated. It varies from country to country and is usually based on relative socioeconomic capacity ^7^. For this review, we will accept and describe the study authors’ definition of deprivation.

### Outcomes

***Main outcome(s)***

The outcomes will be based on the core outcome set for multimorbidity ^10^ that recommends primary outcomes of quality of life, mental health and mortality for interventions focused on multimorbidity.

The primary outcomes for the review will be:

- Health related quality of life (HRQoL) from baseline to last available follow up, as measured by a validated instrument, such as EQ5D5L, SF-12, SF-36 or The World Health Organization Quality of Life (WHOQOL).
- Mental health outcomes from baseline to last available follow up, as measured by a validated instrument such as the Warwick Edinburgh mental wellbeing scale or the Hospital Anxiety and Depression score.

***Additional outcome(s).*** Secondary outcomes measured will also focus on the core outcome set for multimorbidity ^10^. While this is a wide range of outcomes it is in keeping with the MRC frameworks’ guide on using multiple outcome measures for complex interventions ^11^. These include:

- Patient-reported impacts and behaviours will include measures of social-connectedness or isolation, self-rated health, patient experience of care, treatment burden, self-management behaviour and self-efficacy.
- Physical activity and function will include measures of physical activity (self-reported or objectively measured), physical function, activities of daily living.
- Health service utilisation will be defined as number of GP visits, ED attendances or hospital admissions as measured via primary care or hospital records or self reported.
- Any physical health data reported will also be included.

Lack of this data will not exclude studies from the review.

Economic issues will also be considered in the review. This involves the assembly, selection, critical appraisal, summary and synthesis of data from relevant health economics studies. Evidence on marginal resource use and costs associated with an intervention, versus relevant comparators, is important in any decision-making regarding investing in social prescribing. Economic studies linking costs to health and social outcomes will also be reviewed.

## Search strategy

The following bibliographic and trials databases will be searched from inception up to May 2019, with no language limits:

Cochrane database, Cochrane Central register of Controlled trials, ClinicalTrials.gov and EU Clinical Trials Register, Cumulative Index of Nursing and Allied Health Literature (CINAHL), Embase, PubMed/MEDLINE, Psychinfo, LILACS (Latin American and Caribbean Health Sciences Information database), PAHO (Pan American Health Organization database), and Scopus.

To identify economic evaluations that may be of relevance the NHS EED (NHS Economic Evaluation Database), Health Technology Assessment Database (both available via the Centre for Reviews and Dissemination (CRD), University of York) and CEA (Cost-Effectiveness Analysis Registry) will be searched.

Search terms for PubMed will include keywords and phrases in the title and abstract; “link worker”, “social prescri*”, “community health worker*”, “patient navigators”, “health facilitator” and variations of these terms. The terms will be informed by previous reviews and scoping reviews. The strategy will be adapted for specific databases. A key word search will be more specific, as some studies may not be MESH term, or equivalent, indexed. A manual review of titles will exclude obviously irrelevant studies.

A forward and backward citation search of retrieved articles will be conducted for additional relevant literature.

### Grey literature searches

The following databases will be searched: Irish Health Service Executive (HSE) Lenus, RIAN, Open Grey, DART EUROPE, Google and Google Scholar and WHOLIS (World Health Organization Library Information System).

In addition, social prescribing networks will be contacted for grey literature reports and authors of relevant literature directly regarding additional unpublished reports.

### Search strategy example (PubMed)

Please see Extended data for sample search ^12^.

## Data management

Rayyan will be used to sort abstracts for inclusion and exclusion. Revman 5 will be used to store and manage selected articles and manage extracted data. References will be managed with Endnote 8 reference manager.

## Data extraction

The lead author (BK) will do an initial screen to remove clearly ineligible titles. Two independent reviewers will then review all potentially eligible titles and abstracts of the results of the search strategy and select those meeting the review criteria (BK and AC). Any discrepancies will be discussed with a third reviewer (SMS). In cases where it is unclear from the title or abstract whether a study should be included, the full text will be obtained.

After initial selection, the full text of each eligible study will be retrieved and reviewed for final inclusion by two reviewers (BK and AC). Any discrepancies will be resolved through discussion with a third reviewer (SMS). Reasons for exclusion will be documented using the Cochrane Data Extraction and Assessment form (study eligibility) and a Preferred Reporting Items for Systematic Reviews (PRISMA) flow diagram will document the selection process ^13^.

A standardised, pre-piloted form will be used to extract data from the included studies for evidence synthesis (Extended data ^12^). Extracted information will include: study setting; study population and participant demographics and baseline characteristics, in particular if any participants were identified as experiencing multimorbidity or social deprivation, details of the intervention to include link worker definition, training, setting and duration of link worker support, details of control conditions; study methodology; recruitment and study completion rates; outcomes and times of measurement; any costs and marginal resource use reported.

## Quality assessment

The included studies will be assessed for bias using the Cochrane Risk of Bias tools for RCTs and nRCTs ^14^. Performance bias will be inherent in all studies as blinding of participants is not possible due to the nature of the intervention. Publication bias will be assessed using a funnel plot if greater than 10 studies are identified. The GRADE assessment tool will be used to rate the quality of scientific evidence and present the evidence summary for each outcome including relative and absolute effects, patient numbers, quality of evidence and why this rating was applied ^15^. 

The health economist (E O’S) will advise on the relevant use of proxy outcomes for economic comparisons, whether validated tools have been used and if so have they been used as intended; the necessary and appropriate use of assumptions and their validity (e.g. whether inferences in SROI models are clearly stated and justified); whether steps have been taken to mitigate the effects of potential confounding factors.

## Strategy for data synthesis

A narrative synthesis will be performed and presented in tabular form to include the following headings: study method, nature of intervention, number of participants, outcome measures used, effects, costs and cost effectiveness implications. Studies that have calculated the social return on investment (SROI) will also be examined.

A statistician will advise on whether there is sufficient comparable data to conduct a meta-analysis of the effect size of the HRQOL and mental health and wellbeing measures. RevMan ^16^ or Stata version 15 ^17^ will be used. 

For continuous variables, standardised mean differences (SMD) will be estimated, with adjustment for the direction of the scale. Conventionally, SMD values of 0.2, 0.5 and 0.8 are taken as small, medium and large effect sizes, respectively ^18^. Dichotomous outcomes, if any, will be presented as risk ratios (RR) or odds ratios (OR) with 95% confidence intervals. Studies will be pooled together and analysed using a random-effects (RE) model to obtain the summary effect estimate, 95% confidence interval and p-value. Studies using binary and continuous outcomes will be analysed separately. Heterogeneity between studies will be explored through visual inspection of the forest plots and using the *I ^2^* statistic. We will interpret an *I ^2^* value of 0% as an indication of no observed inconsistency/heterogeneity, 30%–60% as may represent moderate heterogeneity, 50%–90% as may represent substantial heterogeneity and 75% to 100% considerable heterogeneity ^19^. 

Where required study data is incomplete or clarifications are needed, authors will be contacted. Following contacting authors, if data is still missing, estimation of standard deviations (SDs) will be done by borrowing SDs from other studies included in this meta-analysis.

If ten or more trials are included in the meta-analysis, a funnel plot and Egger’s test will be used to assess publication bias.

The following sensitivity analyses will be conducted: excluding high risk of bias studies, as classified under ‘risk of bias’ assessment, and excluding outcomes with imputed values.

## Analysis of subgroups

If sufficient data are available from studies with comparable interventions and outcomes, sub-group analyses of participants with multimorbidity, living in areas of social deprivation and both will be completed. This will be a narrative synthesis and will include a meta-analysis if sufficient data are available. Similarly, studies with link workers based in primary care practices vs those with a link worker based in the community will be compared if sufficient studies are identified.

## Dissemination of information

The review will be published in a peer-reviewed journal, reported using the PRISMA guidelines ^13^. The review will also be presented at a relevant conference and disseminated to policy-makers, patients, and the public

## Study status

Database searches have been completed and title and abstract review is underway.

## Data availability

### Underlying data

No data is associated with this article.

### Extended data

Open Science Framework: Effectiveness of link workers providing social prescribing on health outcomes and costs for adult patients in primary care and community settings. A protocol for a systematic review of the literature. Extended Data. https://doi.org/10.17605/OSF.IO/X6V2K ^12^

This project contains the following extended data:

- Pubmed Search Strategy for Effectiveness of link workers systematic review.docx (PubMed search strategy)
- Data extraction pilot template.xlsx (Spreadsheet containing the study data extraction form)

### Reporting guidelines

PRISMA-P checklist for “Effectiveness of link workers providing social prescribing on health outcomes and costs for adult patients in primary care and community settings. A protocol for a systematic review of the literature” https://doi.org/10.17605/OSF.IO/X6V2K ^12^

Data are available under the terms of the Creative Commons Zero “No rights reserved” data waiver (CC0 1.0 Public domain dedication).
