# Supplementary material for: Effectiveness of link workers providing social prescribing on health outcomes and costs for adult patients in primary care and community settings. A protocol for a systematic review of the literature
Source: HRB Open Res. 2020 Nov 17;2:21. Originally published 2019 Sep 2. [Version 2] doi: 10.12688/hrbopenres.12936.2 (PMC7745185; doi:10.12688/hrbopenres.12936.2)
Supplement: Peer review history

# Peer review: Reviewer response for version 2

**DOI:** 10.21956/hrbopenres.14321.r28386

**Published:** 2020-12-08

**Authors:** Mark Fort Harris

**Affiliations:** 1 Centre for Primary Health Care and Equity, University of New South Wales, Sydney, Australia

No new comments. The authors have adequately addressed my previous points.

Is the study design appropriate for the research question?

No

Is the rationale for, and objectives of, the study clearly described?

Yes

Are sufficient details of the methods provided to allow replication by others?

Partly

Are the datasets clearly presented in a useable and accessible format?

Partly

Reviewer Expertise:

Primary Health Care, Health Equity.

I confirm that I have read this submission and believe that I have an appropriate level of expertise to confirm that it is of an acceptable scientific standard.

# Peer review: Reviewer response for version 2

**DOI:** 10.21956/hrbopenres.14321.r28385

**Published:** 2020-11-17

**Authors:** Mark Petticrew

**Affiliations:** 1 Department of Public Health, Environments and Society, London School of Hygiene and Tropical Medicine (LSHTM), London, UK

No further comments. This will be an important and valuable review.

Is the study design appropriate for the research question?

Yes

Is the rationale for, and objectives of, the study clearly described?

Yes

Are sufficient details of the methods provided to allow replication by others?

Yes

Are the datasets clearly presented in a useable and accessible format?

Yes

Reviewer Expertise:

Public Health.

I confirm that I have read this submission and believe that I have an appropriate level of expertise to confirm that it is of an acceptable scientific standard.

# Peer review: Reviewer response for version 1

**DOI:** 10.21956/hrbopenres.14016.r27556

**Published:** 2020-07-06

**Authors:** Mark Fort Harris

**Affiliations:** 1 Centre for Primary Health Care and Equity, University of New South Wales, Sydney, Australia

This is a generally well described protocol for a systematic review on link workers social prescribing in primary care and community settings.

 There are a few areas that need clarification:

1. The definition of LW is under intervention - this could be more clearly positioned in the paper.
2. The definition says that “link workers are non-health or social care professions based in primary care practices or community and/or voluntary organisations..”.  However use of volunteers as link workers is excluded.  Also the search terms include “community health workers”, “patient navigators” and “health facilitator” - aren’t these are health professionals?
3. Disability support workers are not included in the search terms.
4. Interventions for specific conditions will be excluded.  Does this mean that interventions for specific groups of patients (e.g. supporting diabetes patients to apply for financial support for that condition)?
5. Why are mental health outcomes the only specific conditions for which outcomes will be assessed?

Is the study design appropriate for the research question?

No

Is the rationale for, and objectives of, the study clearly described?

Yes

Are sufficient details of the methods provided to allow replication by others?

Partly

Are the datasets clearly presented in a useable and accessible format?

Partly

Reviewer Expertise:

Primary Health Care, Health Equity.

I confirm that I have read this submission and believe that I have an appropriate level of expertise to confirm that it is of an acceptable scientific standard, however I have significant reservations, as outlined above.

# Response

**Published:** 2020-11-13

**Authors:** Bridget Kiely

**Affiliations:** Dr, Ireland

Many thanks for this helpful and detailed review. Apologies for the delay in response. The lead author has just returned from maternity leave. We have submitted a revised version of the manuscript, which we hope will address the points you have raised and provide greater clarity for readers. Please also see individual response below. 

 1. The definition of LW is under intervention - this could be more clearly positioned in the paper.

**Author Response**

 We have now repositioned the full definition of link workers to the beginning of the paper, within the Introduction in paragraph 1.

 2. The definition says that “link workers are non-health or social care professions based in primary care practices or community and/or voluntary organisations..”.  However use of volunteers as link workers is excluded.  Also the search terms include “community health workers”, “patient navigators” and “health facilitator” - aren’t these are health professionals?

**Author Response**

 We have expanded the inclusion and exclusion section to make this clearer. For the purpose of this review we are not considering volunteers as professionals. We are making a distinction between interventions where support is provided by a health and social care professional (someone with a recognised health or social work professional qualification) versus a “lay worker” who may be working in the area of health, potentially have a related qualification, but is not a member of a recognised health or social work professional body.

  3. Disability support workers are not included in the search terms.

**Author Response**

 While disability support workers also engage in elements of social prescribing and health and social care coordination, as they also provide personal care services it would be difficult to disentangle the impact of this on outcomes and so this was not included in the search terms. We have added a clarification to the exclusion criteria.

  4. Interventions for specific conditions will be excluded.  Does this mean that interventions for specific groups of patients (e.g. supporting diabetes patients to apply for financial support for that condition)?

**Author Response**

 The inclusion and exclusions section has been adjusted to make this clearer, within the Methods under Types of Interventions. There are a lot of interventions that use lay workers to support people with specific diseases. Many such interventions may include some elements of social prescribing, but the majority of the intervention focuses on disease education and support around disease control such as medication adherence. As such it is hard to disentangle the impact of the different components on outcomes and so such disease specific interventions have been excluded.

  5. Why are mental health outcomes the only specific conditions for which outcomes will be assessed?

**Author Response**

 Mental health outcomes are one of the primary outcomes recommended by the Core Outcome Set for Multimorbidity, which provides our outcomes framework. A recent literature review of the aims of social prescribing programmes found that improved mental health was the most common, although the programmes were not specifically aimed at people with mental health problems  (Rempel ES, Wilson EN, Durrant H, et al

 Preparing the prescription: a review of the aim and measurement of social referral programmes

 BMJ Open 2017;7:e017734. doi: 10.1136/bmjopen-2017-017734) Mental health outcomes such as anxiety or depression are often reported for all individuals in a study regardless of a diagnosis of a mental health condition. To clarify, we are not specifically including mental health conditions and all outcomes reported using a validated measure will be considered. 

# Peer review: Reviewer response for version 1

**DOI:** 10.21956/hrbopenres.14016.r26866

**Published:** 2019-10-25

**Authors:** Mark Petticrew

**Affiliations:** 1 Department of Public Health, Environments and Society, London School of Hygiene and Tropical Medicine (LSHTM), London, UK

This is generally very clear and likely to be a well-conducted review. A few minor comments:

 The selection of outcomes could be clearer. Much of the Introduction section (appropriately) discusses a potentially wide range of outcomes from the use of link workers. However not all of these appear in the list of primary or additional outcomes. Some of the omissions seem to be important - e.g. link workers may facilitate health and social care co-ordination but this does not appear in the additional outcomes. Also, the primary outcomes are QoL and mental health, but surely (going by the introduction) physical health outcomes are also relevant primary outcomes.

 Another potentially important outcome relates to inequalities  - i.e the differential effects of link workers in different groups  - (e.g. this could be analysed by stratifying the findings by SES, gender etc if possible, or some other form of subgroup analysis) it would be useful to explore this in the review.

Is the study design appropriate for the research question?

Yes

Is the rationale for, and objectives of, the study clearly described?

Yes

Are sufficient details of the methods provided to allow replication by others?

Yes

Are the datasets clearly presented in a useable and accessible format?

Yes

Reviewer Expertise:

Public Health.

I confirm that I have read this submission and believe that I have an appropriate level of expertise to confirm that it is of an acceptable scientific standard.

# Response

**Published:** 2020-11-13

**Authors:** Bridget Kiely

**Affiliations:** Dr, Ireland

Thank you for the helpful and positive review and suggestions for clarifications.  Apologies for the delay in responding. There was a delay in obtaining a second review and the lead author has just returned from maternity leave.

 1. The selection of outcomes could be clearer. Much of the Introduction section (appropriately) discusses a potentially wide range of outcomes from the use of link workers. However not all of these appear in the list of primary or additional outcomes. Some of the omissions seem to be important - e.g. link workers may facilitate health and social care co-ordination but this does not appear in the additional outcomes. Also, the primary outcomes are QoL and mental health, but surely (going by the introduction) physical health outcomes are also relevant primary outcomes.

**Author Response**

 The selection of outcomes is based on the Core Outcome Set for Multimorbidity as multimorbidity is a specific focus of this review. The COSmm  recommends quality of life, mental health and mortality as primary outcomes. Secondary outcomes are grouped into patient reported impacts and behaviour, physical activity and function, consultation related and health service utilisation. We do not anticipate that link workers connecting people to community resources will have significant consultation related outcomes and so have not listed this specifically. The COSmm is broadly in line with the outcome measures suggested in the NHS draft Core Outcomes Framework for Social Prescribing, that mentions mental health and wellbeing scales, physical activity measures, patient activation measures, social isolation measures as well as health care utilisation measures. While link workers can indeed provide health and social care coordination this is one of the range of ways in which they can support clients in order to improve outcomes such as quality of life and mental health. We have not therefore included this as a separate outcome but it would be captured by a patient experience of care measure if reported in included studies and we have specifically added this as a secondary outcome. 

 The listed outcomes are not finite and we intend to include all reported outcomes, including physical health measures, that are reported using a validated measure. The text has been adjusted to make the selection of outcomes clearer.

 2. Another potentially important outcome relates to inequalities  - i.e the differential effects of link workers in different groups  - (e.g. this could be analysed by stratifying the findings by SES, gender etc if possible, or some other form of subgroup analysis) it would be useful to explore this in the review.

**Author Response**

 We agree that link workers have the potential to reduce health inequalities. People in areas of social deprivation experience more multimorbidity at a younger age and have worse outcomes than those in less deprived areas. We intend to do sub group analysis of participants with multimorbidity and those living in areas of social deprivation if there are sufficient studies with data and comparable outcomes. This will provide more evidence on the effectiveness of link workers in areas of deprivation and for people with multimorbidity.
